# Osteoclast inhibitor treatment among men with metastatic castration-resistant prostate cancer

**DOI:** 10.31487/j.COR.2018.03.001

**Published:** 2018-09-06

**Authors:** Daniel Sonnenburg, Parul Chaudhuri, Amy J. Graves, David F. Penson, Alicia K. Morgans

**Affiliations:** 1 Northwestern University Feinberg School of Medicine, Chicago, IL; 2 Vanderbilt University School of Medicine, Nashville, TN; 3 Vanderbilt University Center for Quantitative Sciences and Department of Biostatistics, Nashville, TN

## Abstract

**Background:**

National Comprehensive Cancer Network guidelines recommend monthly osteoclast inhibitor treatment (OIT) in men with metastatic castration-resistant prostate cancer (mCRPC) to prevent skeletal related events (SREs). We assessed adherence to guidelines by quantifying treatment for SRE prevention in a population-based cohort of men with mCRPC.

**Methods:**

Using Surveillance, Epidemiology, and End Results-Medicare data, we identified men aged >65 with prostate cancer as a primary cause of death during 2006–2010. We assessed OIT during a 12-month period between 15 and 3 months before death and used multivariable negative binomial regression to identify factors associated with treatment.

**Results:**

Among 9,634 men who died of prostate cancer, 22% received ≥ 1 OIT, and use increased slightly over time. Men age 75–84 and ≥ 85 were less likely than younger men to be treated (IRR 0.63, 95% CI 0.49–0.78 and IRR 0.34, 95% CI 0.17–0.50, respectively). African American men were less likely than white men to receive OIT (IRR 0.75, 95% CI 0.54–0.95), as were men from areas with lower median income (P=0.014). Compared with men seeing a urologist only, men seeing a medical oncologist and a urologist (IRR 2.52, 95% CI 2.36–2.68) or a medical oncologist alone (IRR 3.82, 95% CI 3.54–4.09) had higher incidence rates of treatment.

**Conclusions:**

Fewer than a quarter of American men dying of prostate cancer received recommended treatment to prevent SREs within the final year of their lives, with particularly low rates of treatment among older men, African American men, and those living in areas with low median income. Visits with a medical oncologist were associated with increased use. Further evaluation of these disparities by age, race and socioeconomic status are necessary to identify interventions to reduce them.

## Introduction

Prostate cancer is the most common non-cutaneous malignancy among American men and is the second leading cause of cancer death in the United States [1]. Men with metastatic prostate cancer are at risk for skeletal related events (SREs) from cancer treatment-induced bone loss, pathologic fractures, and pain from progression of prostate cancer [2]. Skeletal related events are a leading cause of morbidity and increased mortality among men with prostate cancer [2]. Osteoclast inhibitor treatment (OIT) reduces the risk of SRE, hospitalization, and mortality in men with prostate cancer [3]. National guidelines include a category 1 recommendation recommending up to monthly treatment of men with metastatic castration-resistant prostate cancer (mCRPC) with zoledronic acid or denosumab to reduce the risk of SREs in this high-risk population [4].

Whether clinical practice in the United States adheres to the guideline recommendations for monthly treatment with OIT has not been reported. We hypothesized that there would be overall low utilization of OIT, and that rates of treatment would be associated with identifiable clinical and sociodemographic factors. We assessed rate of treatment with OIT in a large, population-based cohort of older American men with prostate cancer during a 12-month period beginning 15 months before death and concluding 3 months before death from prostate cancer. We also identified patient, physician, and disease factors associated with treatment with bisphosphonates. The analysis was restricted to bisphosphonate utilization due to years of SEER-Medicare data available.

## Materials and Methods

### Data

We used Surveillance, Epidemiology and End Results (SEER) Medicare data for this investigation. The National Cancer Institute’s SEER registry program collects data, including patient sociodemographics, tumor characteristics, and treatment information, for each incident cancer identified in a region. These population-based cancer registries currently reflect approximately 28% of the United States population [5]. SEER data and Medicare administrative data have been merged using a matching algorithm that links files for over 94% of SEER patients aged 65 or older [6]. The Medicare claims data used in this study included the Medicare Provider Analysis and Review file (inpatient admissions), the 100% Physician/Supplier file (physicians’ services for comorbidity assessment and ascertainment of bone density testing and ADT), and the Hospital Outpatient Standard Analytic file (outpatient facility services to identify comorbidity and bone density testing and ADT).

### Study Cohort

We identified all men 65 years of age or older enrolled in parts A and B of fee-for-service Medicare with prostate cancer as a primary cause of death during the period of 2006–2009 (N = 9634). Men with an International Classification of Diseases, ninth edition [ICD-9] diagnosis codes 185.xx (Malignant neoplasm of prostate) and V10.46 (Personal history of malignant neoplasm of prostate) were included. Medicare lacks a diagnosis code for CRPC, and metastatic disease is coded inconsistently. Because a large majority of men dying of prostate cancer have castration-resistant disease, and because 90% of men dying of prostate cancer have bone metastases, the identified population with prostate cancer identified as a primary cause of death should predominantly include men with CRPC with bone metastases in the 15 months prior to death [1].

### Receipt of OIT

We measured receipt of available OIT during the time period, including zoledronic acid, pamidronate, due to the database years available for analysis. We described receipt of OIT during a 12-month period beginning 15 months before death and concluding 3 months before death when care intensity may decrease due to enrollment on hospice. We limited the expected period of hospice enrollment to a 3-month period prior to death because very few individuals are enrolled in hospice for a prolonged period prior to death from cancer, with <10% patients utilizing hospice for > 6 months at the end of life [7]. Health Care Common Procedure Coding System [HCPCS] codes used to track OIT administration included J3487, J3488, Q4095, J2430, C9411, C9272, J3590, as well as ICD-9 code E933.6.

### Patient Characteristics

We characterized patients’ age, race/ethnicity, marital status, SEER region, comorbid illness at the time of diagnosis (based on the Klabunde modification of the Charlson Index), year of diagnosis, stage at diagnosis, tumor grade (by Gleason score), primary treatment (surgery, radiation, or neither), median household income, proportion of high school graduates in the census tract of residence (categorized in quartiles within registries), and year of death [8, 9]. We also characterized visits with urologists and medical oncologists, as they are the providers most likely to treat men with mCRPC with bisphosphonates, and identified men seen only by other types of providers. Variables were categorized as in Table 1.

### Analyses

We used negative binomial regression with an offset of log follow-up time to identify factors associated with treatment with bisphosphonates, including patient and tumor characteristics, and the physicians with whom they had outpatient visits. Independent variables included all variables in Table 1. All tests of statistical significance were two sided. We used R statistical software for analyses. The study was approved by the institutional review board at Vanderbilt University Medical Center.

### Results

Characteristics of the 9,634 men who died of prostate cancer during the study period are included in Table 1. Overall, 2,094 (22%) received treatment with at least one dose of OIT during the 12-month study period (Table 1), and 2,364 (25%) experienced a skeletal related event (Table 1).

**Figure 1:**
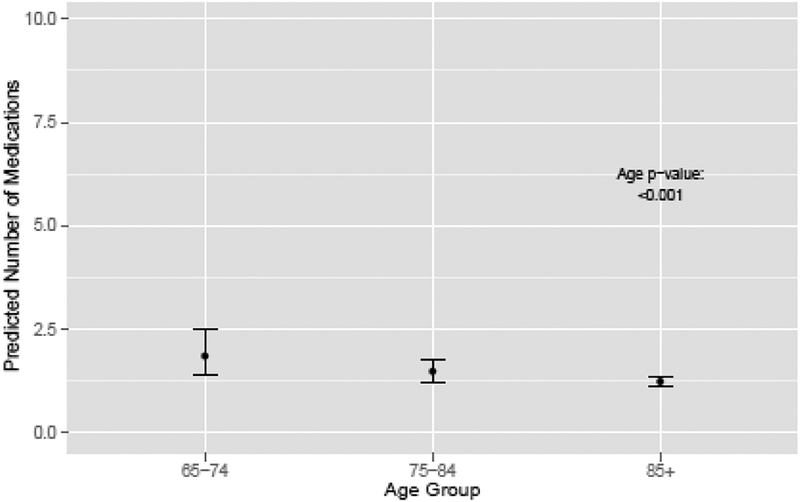
Predicted number of medications by age group

**Figure 2:**
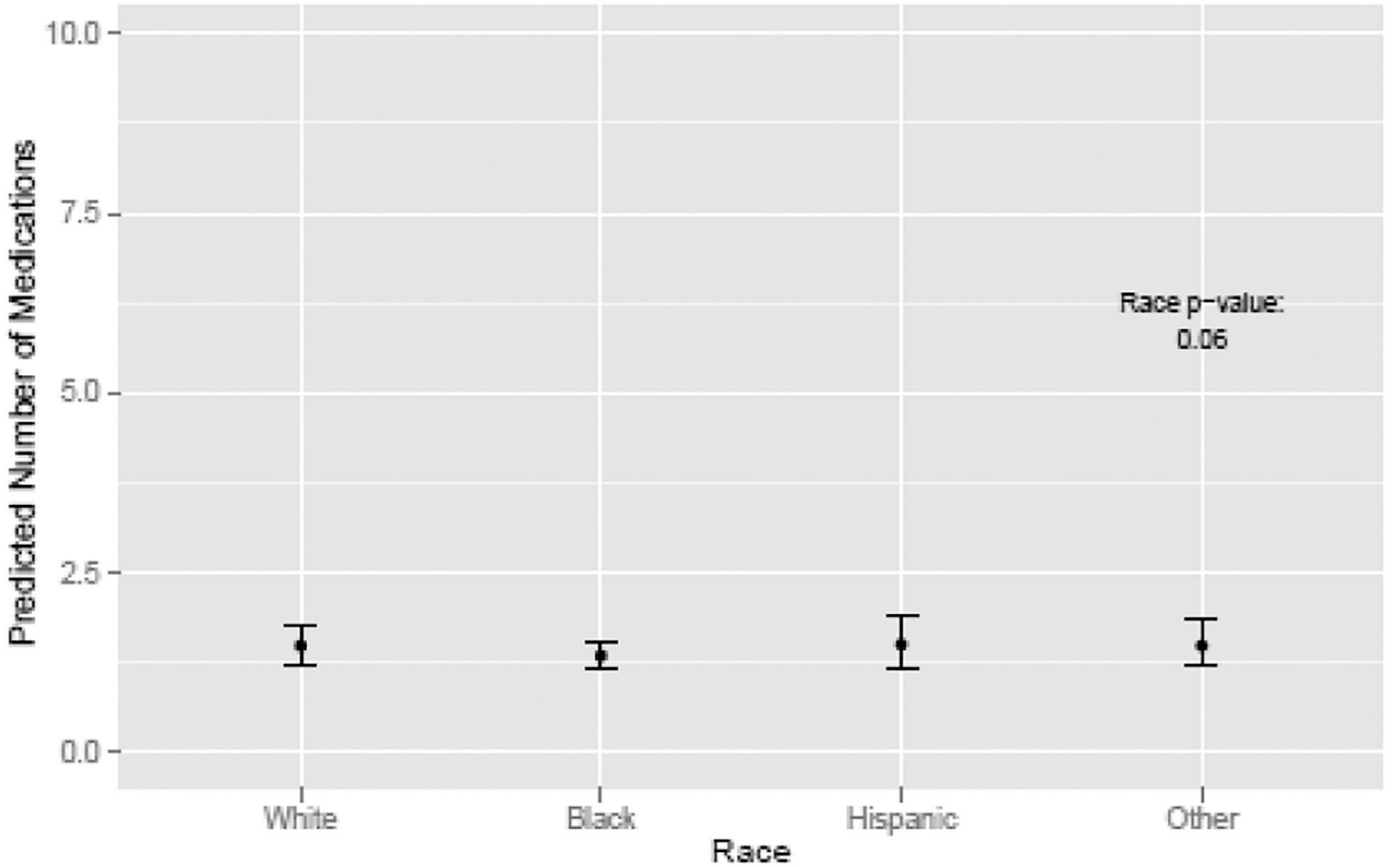
Predicted number of medications by race

Unadjusted rates of treatment by patient characteristics are presented in Table 1 with incidence rate ratios [IRR] and 95% confidence intervals [CIs]. The likelihood of treatment with OIT decreased with increasing age (Figure 1). Men ≥85 years old and men 75–84 years old were less likely to receive treatment than men age 65–74 (IRR 0.34, 95% CI 0.17­0.50 for men ≥ 85, and IRR 0.63, 95% CI 0.49–0.78) (Table 1). OIT rates varied by race (Figure 2). African American men were less likely than white men to receive treatment (IRR 0.75, 95% CI 0.54–0.95), but there was no significant difference in treatment rates between other races and white men (Table 1). Men living in areas of the two highest quartiles of median income were more likely to receive OIT than men in areas of the lowest income quartile (IRR 1.32, 95% CI 1.14–1.51 for quartile 3, and IRR 1.31, 95% CI 1.09–1.53 for quartile 4). Men diagnosed in later years of the study were less likely to receive OIT than men diagnosed in 1995 (Table 1). The likelihood of OIT did not vary significantly by comorbidity burden, education level, SEER region, or Gleason score (Table 1).

OIT was more likely among several populations. Married men were more likely to receive treatment than unmarried men (IRR 1.39, 95% CI 1.26–1.52). Men with metastatic disease at the time of diagnosis were more likely to receive OIT than men who were initially diagnosed with localized disease (IRR 1.99, 95% CI 1.84–2.13). The likelihood of OIT increased yearly with men dying later in the study having a higher likelihood of treatment than men dying in 2006–2007 (Table 1). Men treated with ADT (androgen deprivation therapy) via orchiectomy or GnRH (gonadotropin-releasing hormone) agonist were more likely to OIT than men not treated with ADT, and men with osteoporosis were more likely to receive treatment than men without osteoporosis (IRR 3.18, 95% CI 3.04–3.31 for men treated with a GnRH agonist, IRR 1.83, 95% CI 1.44–2.23 for men treated with orchiectomy, and 1.24, 95% CI 1.05–1.44 for men with osteoporosis). The unadjusted likelihood of OIT also varied substantially by SEER region, with highest rates in Greater California and New Jersey, and the lowest rates in Rural Georgia and Hawaii (Table 1).

**Figure 3:**
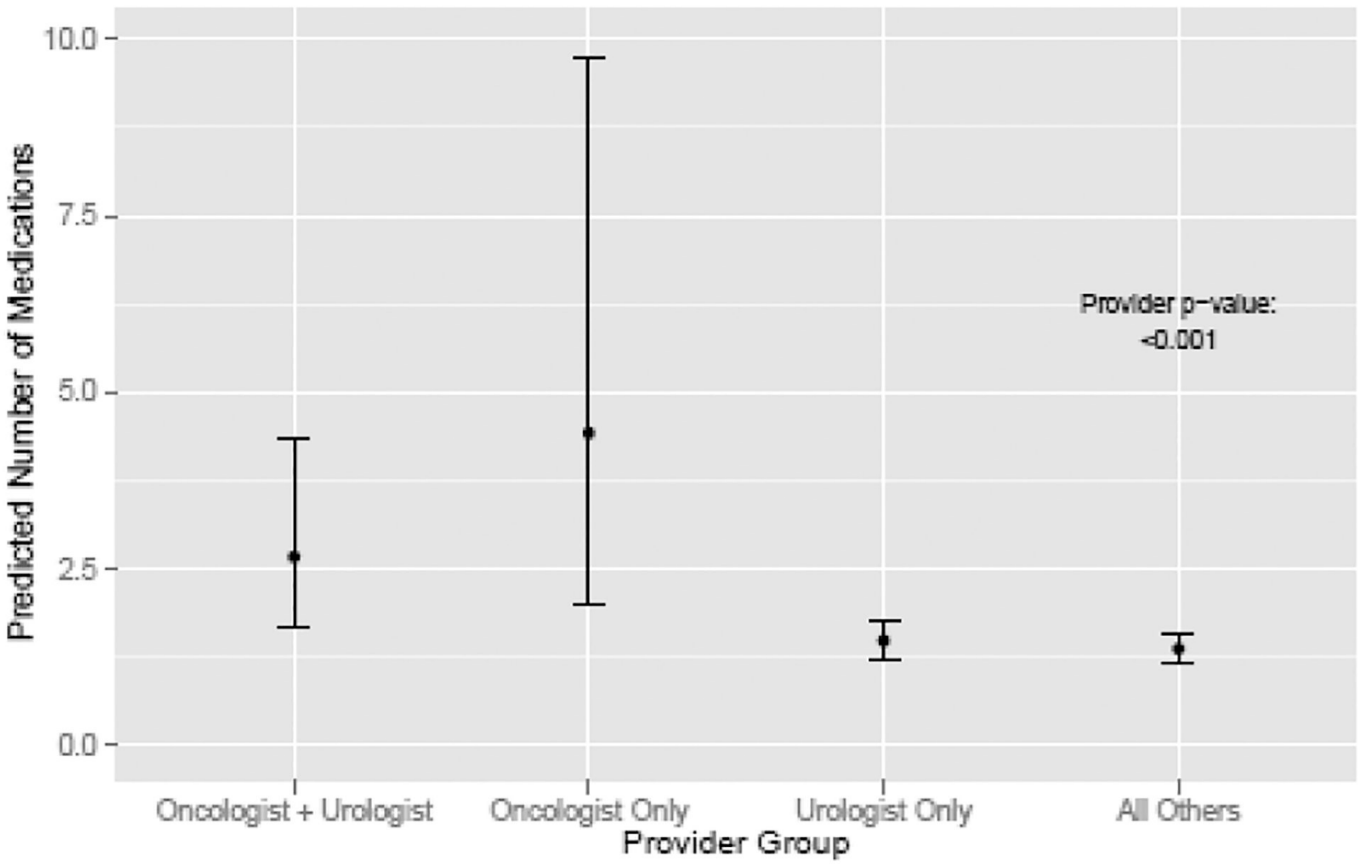
Predicted number of medications by provider group

The types of physicians with whom patients had visits were also associated with treatment (Figure 3). Men seeing a medical oncologist were more likely than men seeing only a urologist to receive treatment (IRR 3.82, 95% CI 3.54–4.09 for men seeing a medical oncologist only, and IRR 2.52, 95% CI 2.36–2.68 for men seeing both a medical oncologist and a urologist) (Table 1). Men only seeing other types of physicians were less likely to receive OIT than men seeing a urologist only (0.79, 95% CI 0.62–0.96) (Table 1).

### Discussion

We evaluated the rate of OIT in a population-based cohort of men with advanced prostate cancer between 2006 and 2009. During the study period, only 22% of men received at least one dose of OIT, and 25% experienced a clinically relevant SRE. Several populations, including African American men and elderly men, and men living in areas of low median income, were significantly less likely to receive treatment than Caucasians, younger men, and men living in areas of higher median income, respectively. Treatment by a medical oncologist was associated with greater incidence of OIT than treatment by a urologist alone.

Our observation that African American men had lower rates of OIT may be related to the fact that African American men generally have a higher baseline bone mineral density than Caucasian men [10]. A previous study demonstrated that African American men with prostate cancer are also less likely to undergo bone density testing, possibly reflecting knowledge of greater bone mineral density in this population versus a disparity in the provision of screening and supportive care for these patients [11]. Elderly men received OIT less commonly than younger patients, identifying a disparity that is particularly striking as this population has greater rates of osteoporosis and falls as compared with younger men. Finally, we identified difference in physician specialty and treatment, likely due to zoledronic acid requiring intravenous infusion. Should this analysis be repeated in a more contemporary cohort, we expect that differences between specialties would decrease as practices are able to administer denosumab via subcutaneous injection rather than requiring an infusion center for administration.

Our study is the first to report on rates of treatment with OIT in a nationally-representative population-based cohort of men with mCRPC. Previous studies describing OIT utilization in men with metastatic prostate cancer report similarly low rates of bisphosphonate utilization [12, 13]. A series of 147 chart reviews from men with confirmed mCRPC treated in one of 15 community-based urology practices reported that 49% of patients received at least one dose of bisphosphonates [12]. The higher rate of treatment in this group may be due to the small number of select practices included. Similar to our analysis, a separate study of 461 patients with bone metastatic prostate cancer enrolled in one of two private US health care systems found that only 20.2% of men received treatment with bisphosphonates [13]. The differences between these studies are likely due to sample selection and size, with this study more likely representing utilization rates nationally given the cohort and size of the study.

While our study reports multiple clinically relevant findings, we acknowledge that it has several limitations. Limited clinical data were available in SEER including lack of a diagnosis code for mCRPC and we defined our cohort by a series of assumptions that may not include all patients who have mCRPC. To address these, we performed sensitivity analyses in a cohort defined by having a diagnosis of prostate cancer, death from prostate cancer, and a code for bone metastases, and our results were unchanged. Further, code-based population studies do not include patient-level data on potentially relevant variables such as performance status, dental history, or information related to treatment decisions between patients and physicians. Additionally, the 12-month window of observation would not capture receipt of bisphosphonates outside of the window. Given the guideline recommendation for monthly treatment with OIT, however, we believe that identifying at least one treatment during the 12-month period was reasonable to inform our understanding of practice patterns. Finally, this dataset does not include the most contemporary rates of utilization due to availability of datasets and follow up studies are necessary to define utilization rates of newer osteoclast targeted agents such as denosumab. We postulate that rates of OIT will be higher in the era of denosumab as physicians can administer it without an infusion center, and differences in utilization by specialty are also likely less pronounced. However, this is less likely to affect disparities that exist in OIT by race and age.

## Conclusion

Guideline recommendations based on level one evidence suggest monthly treatment of men with mCRPC with OIT to prevent SREs and reduce morbidity and mortality in this population [14]. Despite this, less than a quarter of American men dying of prostate cancer received OIT in this study. Factors associated with a lower incidence of OIT, including older age, African American race, and lower socioeconomic status, reveal disparities which should be addressed to improve outcomes for men with mCRPC. Follow up studies including interventions targeting the groups with lower rates of treatment identified in this study are necessary to optimally reduce skeletal complications among men with mCRPC.

## Figures and Tables

**Table 1:** Patient characteristics and receipt of bisphosphonates during 12-month period beginning 15 months before death from prostate cancer.

	N (%)	% who received bisphosphonate	P value*	Incidence Rate Ratio	95% Confidence Interval	P value**
Total	9634 (100)	21.7				
						
Age in years			<.001			
65–74	2508 (26)	8.6		1.00		Ref
75–84	4222 (44)	9.7		0.63	0.49–0.78	<0.001
≥85	2904 (30)	3.4		0.34	0.17–0.50	<0.001
						
Race			<.001			
Non-Hispanic white	7796 (81)	18.1		1.00		Ref
Non-Hispanic African American	1239 (13)	2.2		0.75	0.54–0.95	0.005
Hispanic	204 (2)	0.5		1.03	0.62–1.45	0.879
Other	383 (4)	0.9		1.00	0.67–1.33	0.998
Unknown	12 (<1)	<0.1				
						
Marital status			<.001			
Unmarried	3001 (31)	4.9		1.00		Ref
Married	5665 (59)	14.8		1.39	1.26–1.52	<0.001
Unknown	968 (10)	2.0				
						
Year of Death			0.5256			
2006–2007	2296 (24)	5.1		1.00		Ref
2007–2008	2455 (25)	5.6		1.12	0.95–1.29	0.1962
2008–2009	2490 (26)	5.4		1.38	1.20–1.55	<0.001
2009–2010	2393 (25)	5.6		1.88	1.70–2.07	<0.001
						
Year of Diagnosis			<.001			
1995	303 (3)	0.6		1.00		Ref
1996	302 (3)	0.8		1.17	0.71–1.63	0.500
1997	298 (3)	0.8		1.31	0.85–1.77	0.249
1998	294 (3)	0.9		1.28	0.82–1.75	0.289
1999	370 (4)	1.2		1.52	1.08–1.96	0.060
2000	707 (7)	1.9		1.28	0.89–1.68	0.218
2001	717 (7)	1.9		1.02	0.62–1.42	0.907
2002	806 (8)	2.1		1.06	0.67–1.46	0.764
2003	797 (8)	2.1		0.85	0.45–1.25	0.424
2004	821 (9)	2.5		1.01	0.61–1.41	0.955
2005	1035 (11)	2.6		0.79	0.40–1.18	0.244
2006	1250 (13)	2.2		0.55	0.16–0.94	0.002
2007	913 (9)	1.2		0.39	−0.02–0.80	<0.001
2008	642 (7)	0.6		0.22	−0.23–0.67	<0.001
2009	379 (4)	0.2		0.18	−0.35–0.71	<0.001
						
SEER Region			<.001			
San Francisco	386 (4)	1.1		1.00		Ref
Los Angeles	716 (7)	1.9		1.01	0.64–1.38	0.967
Connecticut	814 (8)	2.2		0.95	0.58–1.31	0.767
Detroit	663 (7)	1.6		1.17	0.79–1.55	0.422
Hawaii	132 (1)	0.2		0.67	0.03–1.32	0.228
Iowa	748 (8)	1.4		0.70	0.30–1.10	0.078
New Mexico	333 (3)	0.6		0.86	0.40–1.32	0.518
Seattle	611 (6)	1.7		0.93	0.55–1.31	0.712
Utah	320 (3)	0.7		1.35	0.91–1.80	0.177
Atlanta	291 (3)	0.6		0.99	0.54–1.44	0.956
San Jose/Monterey	208 (2)	0.5		0.62	0.12–1.13	0.064
Rural Georgia	26 (<1)	0.1		0.97	−0.30–2.24	0.962
Greater California	1536 (16)	3.3		1.33	0.99–1.67	0.099
Kentucky	627 (7)	1.0		1.21	0.80–1.61	0.367
Louisiana	485 (5)	0.8		0.77	0.33–1.20	0.224
New Jersey	983 (10)	2.5		1.11	0.74–1.48	0.584
Greater Georgia	755 (8)	1.7		1.43	1.04–1.82	0.069
						
Median household income in census tract of residence			<.001			
Quartile 1 (lowest)	2282 (24)	4.1		1.00		Ref
Quartile 2	2282 (24)	4.6		1.10	0.92–1.28	0.281
Quartile 3	2274 (24)	5.8		1.32	1.14–1.51	0.003
Quartile 4 (highest)	2280 (24)	6.4		1.31	1.09–1.53	0.014
Unknown	516 (5)	0.9				
						
% High school graduates in census tract of residence			<.001			
Quartile 1 (lowest)	2283 (24)	6.5		1.00		Ref
Quartile 2	2281 (24)	5.1		0.90	0.71–1.08	0.230
Quartile 3	2274 (24)	4.7		0.93	0.72–1.13	0.476
Quartile 4 (highest)	2280 (24)	4.6		0.97	0.74–1.20	0.785
Unknown	516 (5)	0.9				
						
Tumor grade (Gleason)			<.001			
Well differentiated (2–4)	127 (1)	0.2		1.00		Ref
Moderately differentiated (5–7)	2317 (24)	4.9		0.89	0.40–1.37	0.630
Poorly differentiated / undifferentiated(8–10)	4587 (48)	12.8		1.16	0.67–1.65	0.547
Unknown	2603 (27)	3.8				
						
Charlson comorbidity score			<0.001			
0	3741 (39)	8.7		1.00		Ref
1	2323 (24)	5.6		1.06	0.91–1.22	0.448
2	1626 (17)	3.7		1.11	0.94–1.29	0.231
≥3	1944 (20)	3.7		0.88	0.71–1.05	0.145
						
Disease Stage at Diagnosis			<.001			
Localized	3721 (39)	7.4		1.00		Ref
Locally Advanced	799 (8)	2.7		1.18	0.98–1.38	0.097
Metastatic	2491 (26)	7.1		1.99	1.84–2.13	<0.001
Unknown	2623 (27)	4.6				
						
Physicians Seen During Study Period			<.001			
Urologist, no PCP or medical oncologist	5357 (56)	9.7		1.00		Ref
Urologist and medical oncologist	1560 (16)	7.1		2.52	2.36–2.68	<0.001
Medical Oncologist only	414 (4)	2.2		3.82	3.54–4.09	<0.001
No urologist, PCP or medical oncologist	2303 (24)	2.7		0.79	0.62–0.96	0.008
						
Osteoporosis Diagnosis			<.001			
No	8678 (90)	2.8		1.00		Ref
Yes	956 (10)	19.0		1.24	1.05–1.44	0.030

* Based on chi-square testing.

** Based on negative binomial regression with offset of log follow-up time to adjust standard errors for clustering within registry, also adjusting for variables in the table.

*** Not reported due to confidentiality issues related to small sample sizes.

SRE=skeletal related event; SEER=Surveillance, Epidemiology, and End Results; Gleason grade 7 was categorized as moderately differentiated before January 1, 2003 and as poorly differentiated as of January 1, 2003.

## References

[1] Howlader N, Noone AM, Krapcho M, Neyman N, Aminou R, (2015) SEER Cancer Statistics Review, 1975–2009 (Vintage 2009 Populations), National Cancer Institute Bethesda, MD http://seer.cancer.gov/csr/1975_2009_pops09/, based on November 2011 SEER data submission, posted to the SEER web site, 2012.

[2] Smith M (2007) Androgen deprivation therapy for prostate cancer: new concepts and concerns. Curr Opin Endocrinol Diabetes Obes 14: 247­254. [Crossref] 17940447 10.1097/MED.0b013e32814db88c PMC3047388

[3] Velde N, Wu E, Guo A, Lu M, Yu A, (2010) The benefits of timely intervention with zoledronic acid in patients with metastatic prostate cancer to bones: a retrospective study of the US Veterans Affairs population. Prostate Cancer Prostatic Dis 14: 79–84. [Crossref] 21173792 10.1038/pcan.2010.49

[4] Mohler J, Armstrong A, Bahnson R, D’Amico A, Davis B, (2016) Prostate Cancer, Version 1.2016. J Natl Compr Canc Netw 14: 19–30. [Crossref] 26733552 10.6004/jnccn.2016.0004

[5] Kohler B, Ward E, McCarthy B, Schymura M, Ries L, (2011) Annual Report to the Nation on the Status of Cancer, 1975–2007, Featuring Tumors of the Brain and Other Nervous System. J Natl Cancer Inst 103: 714–736. [crossref] 21454908 10.1093/jnci/djr077 PMC3086878

[6] Potosky A, Riley G, Lubitz J, Mentenec R, Kessler L (1993) Potential for Cancer Related Health Services Research Using a Linked Medicare-Tumor Registry Database. Med Care 31: 749–756. [Crossref] 8336512

[7] Wang S, Aldridge M, Gross C, Canavan M, Cherlin E, (2016) End-of-Life Care Intensity and Hospice Use. Med Care 54: 672–678. [Crossref] 27111747 10.1097/MLR.0000000000000547 PMC4907842

[8] Charlson M, Pompei P, Ales K, MacKenzie C (1987) A new method of classifying prognostic comorbidity in longitudinal studies: Development and validation. J Chronic Dis 40: 373–383. [crossref] 3558716 10.1016/0021-9681(87)90171-8

[9] Klabunde C, Potosky A, Legler J, Warren J (2000) Development of a comorbidity index using physician claims data. J Clin Epidemiol 53: 1258–1267. [Crossref] 11146273 10.1016/s0895-4356(00)00256-0

[10] Tracy J, Meyer W, Flores R, Wilson P, Hochberg M (2005) Racial Differences in Rate of Decline in Bone Mass in Older Men: The Baltimore Men’s Osteoporosis Study. J Bone Miner Res 20: 1228–1234. [Crossref] 15940377 10.1359/JBMR.050310

[11] Morgans A, Smith M, O’Malley A, Keating N (2013) Bone density testing among prostate cancer survivors treated with androgen-deprivation therapy. Cancer 119: 863–870. [Crossref] 23065626 10.1002/cncr.27830 PMC3671351

[12] Freedland S, Richhariya A, Wang H, Chung K, Shore N (2012) Treatment Patterns in Patients with Prostate Cancer and Bone Metastasis Among US Community-based Urology Group Practices. Urology 80: 293–298. [crossref] 22748612 10.1016/j.urology.2012.04.007

[13] Oster G, Lamerato L, Glass A, Richert-Boe K, Lopez A, (2013) Natural history of skeletal-related events in patients with breast, lung, or prostate cancer and metastases to bone: a 15-year study in two large US health systems. Support Care Cancer 21: 3279–3286. [crossref] 23884473 10.1007/s00520-013-1887-3

[14] National Comprehensive Cancer Network. Prostate Cancer (Version 2 2018).

